# Supplementary material for: Identification of photoperiod-induced specific miRNAs in the adrenal glands of Sunite sheep (Ovis aries)
Source: Front Vet Sci. 2022 Jul 22;9:888207. doi: 10.3389/fvets.2022.888207 (PMC9354845; doi:10.3389/fvets.2022.888207)
Supplement: Supplementary file 1 [file Data_Sheet_1.ZIP › Supplementary file/miRDeep2 file.zip!/miRDeep2 file/Know_Structure/SP42A1/oar-mir-154a.pdf]

miRBase precursor : oar-mir-154a
Total read count : 534
oar-miR-154a-5p read count : 5
oar-miR-154a-3p read count : 529
remaining reads : 0

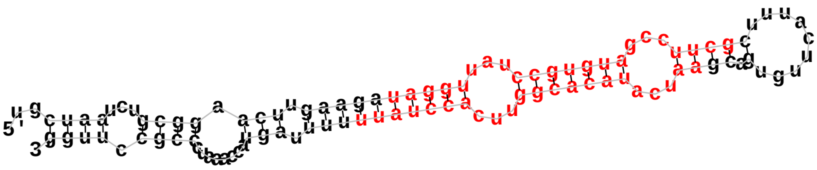

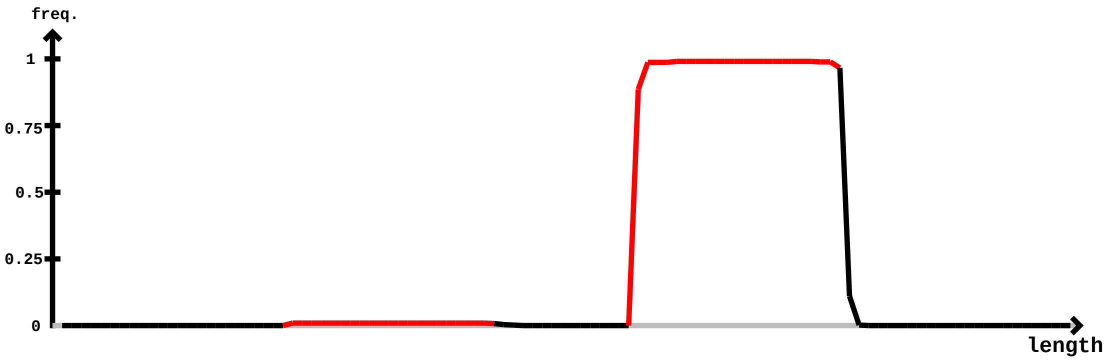

oar-miR-154a-5p

oar-miR-154a-3p

```
5'- ugcuaaucugcggaacuugaagauagguuauccguguagccuucgcuuuacuugugacgaaucauacacgguucaccuauuuuuuaguaccaaaucccgccuugg   -3'    exp
    .((......))((.(((.((((((((((...(((((((...(((((.........).))))...)))))))...)))))))))).))).))..............    reads     mm        sample
    .......................uagguuauccguguagccuuc.............................................................    1         0         seq
    .......................uagguuauccguguagccuucg............................................................    2         0         seq
    .......................uagguuauccguguagccuucgc...........................................................    1         0         seq
    .......................uagguuauccguguagccuucgcu..........................................................    1         0         seq
    ...........................................................aaucauacacgguucaccu...........................    1         0         seq
    ...........................................................aaucauacacgguucaccuau.........................    12        0         seq
    ...........................................................aaucauacacgguucaccuauu........................    442       0         seq
    ...........................................................aaucauacacgguucaccuauuu.......................    18        0         seq
    ............................................................aucauacacgguucaccuauu........................    14        0         seq
    ............................................................aucauacacgguucaccuauuu.......................    39        0         seq
    ............................................................aucauacacgguucaccuauuuu......................    1         0         seq
    ...............................................................auacacgguucaccuauu........................    1         0         seq
    ...............................................................auacacgguucaccuauuu.......................    1         0         seq
```
